# Emergence of *Acinetobacter baumannii* ST730 carrying the *bla*_OXA-72_ gene in Brazil

**DOI:** 10.1590/0074-02760160188

**Published:** 2016-09

**Authors:** Mariana Pagano, Franciéli P Rozales, Diego Bertolini, Lisiane Rocha, Jorge LM Sampaio, Afonso L Barth, Andreza F Martins

**Affiliations:** 1 Universidade Federal do Rio Grande do Sul, Faculdade de Farmácia, Programa de Pós-Graduação em Ciências Farmacêuticas, Porto Alegre, RS, Brasil; 2 Hospital de Clínicas de Porto Alegre, Centro de Pesquisa Experimental, Laboratório de Pesquisa em Resistência Bacteriana, Porto Alegre, RS, Brasil; 3 Laboratório Fleury, Porto Alegre, RS, Brasil; 4 Universidade Federal do Rio Grande do Sul, Programa de Pós-Graduação em Microbiologia Agrícola e do Ambiente, Porto Alegre, RS, Brasil

**Keywords:** Acinetobacter baumannii, MLST, OXA-72

## Abstract

Over the last decade, *Acinetobacter baumannii* resistant to carbapenems has emerged in many medical centres and has been commonly associated with high morbimortality. In Brazil, this resistance is mainly attributed to the spread of OXA-23-producing clones and, to a lesser extent, to OXA-143-producing clones. Here, we describe, for the first time, two OXA-72-producing *A. baumannii* isolates in southern Brazil to a broad spectrum of antibiotics, except polymyxin B and tigecycline. Molecular typing by multilocus sequence typing (MLST) demonstrated that both OXA-72-producing isolates belong to a new sequence type (ST), ST730, which was recently identified in OXA-23-producing *A. baumannii* isolates in São Paulo, Brazil. We demonstrate that the two *A. baumannii* ST730 isolates carrying *bla_OXA-72_*share a common ancestral origin with the *bla_OXA-23_*producers in Brazil. This observation reinforces the importance of strain-typing methods in order to clarify the dynamics of the emergence of new clones in a geographic region.

Recently, a new sequence type, ST730, from an OXA-23-producing *Acinetobacter baumannii* has been deposited in the multilocus sequence typing (MLST) database (http://www.pasteur.fr) (Vasconcelos et al. 2015). In the present study, we describe two isolates of *A. baumannii* ST730 carrying the *bla*_OXA-72_ gene from different patients in a hospital located in southern Brazil.

In March 2013, a carbapenem-resistant *Acinetobacter* sp. was isolated using VITEK^®^2 system (bioMérieux, La Balme-les-Grottes, France) from the tracheal aspirate (> 10^6^ CFU/mL) of a 76-year-old female ICU patient in a 312-bed tertiary care hospital in Porto Alegre, southern Brazil. A few days later, another carbapenem-resistance in *Acinetobacter* sp. was reported from a blood culture (positive in 12 h) of an 86-year-old male ICU patient admitted with sepsis in the same hospital. Both the isolates were identified as *A. baumannii* by matrix-assisted laser desorption/ionisation time-of-flight (MALDI-TOF; Bruker Daltonik, Bremen, Germany) spectrometry and *gyrB* multiplex polymerase chain reaction (PCR). Disc-diffusion assays demonstrated that the isolates were resistant to meropenem, imipenem, amikacin, ampicillin-sulbactam, cefepime and ceftazidime. However, the isolates were found to be susceptible to polymyxin (MICs 1 and 2 µg/mL) and tigecycline (MICs 0.5 and 0.75 µg/mL) as per microdilution and Etest^®^ assays, respectively. The results obtained were interpreted according to the CLSI guidelines (CLSI 2014). The carbapenemase genes (*bla*_OXA-23-like_, *bla*_OXA-24/40-like_, *bla*_OXA-58-like_ and *bla*_OXA-143-like_) were investigated by multiplex PCR as previously described (Higgins et al. 2010). The *bla*_OXA-24/40-like_ gene was detected in both the isolates. Sequencing of *bla*_OXA-24_ gene (ABI 3500 Genetic Analyzer; Applied Biosystems, Foster City, CA, United States) identified the variant *bla*_OXA-72_, which displayed 100% identity to the original *bla*_OXA-72_ gene (GenBank accession number AY739646.1). Clonal diversity, investigated by repetitive-sequence-based PCR (REP-PCR), revealed an identical profile of the isolates (Bou et al. 2000). MLST was performed according to the Institut Pasteur scheme (http://www.pasteur.fr) and both the isolates were identified as ST730.

Resistance to carbapenems among *A. baumannii* isolates from Brazil has been mostly related to the production of OXA-23, followed by OXA-143 (Vasconcelos et al. 2015). In fact, OXA-72-producing *A. baumannii* isolates are still uncommon in Brazil. The first two cases of *A. baumannii* carrying *bla*_OXA-72_ gene were reported from São Paulo (Southeast Brazil) in 2011 (Antonio et al. 2011, Werneck et al. 2011). Two years later, this gene was reported in two other *A. baumannii* isolates from Recife (Northeast Brazil) (Cavalcanti et al. 2013). Recently, a surveillance study evaluated nine hospitals from five different states, representative of all the Brazilian regions, and described an inter-hospital dissemination of 10 *A. baumannii* isolates containing *bla*_OXA-72_ in São Paulo (Vasconcelos et al. 2015). These data highlight the possibility of the spread of this gene in the country. Furthermore (Werneck et al. 2011), reported the presence of *bla*OXA-72 gene inserted on a plasmid of ~ 86 kb, highlighting its potential for spread.

Here, we describe, for the first time, two *A. baumannii* isolates harbouring the *bla*_OXA-72_ gene isolated from Rio Grande do Sul, southern Brazil. These data point towards the increasing diversity of oxacillinases among the clinical isolates of *Acinetobacter* spp. in Brazil. Recently, Cayô et al. (2015) deposited an OXA-23-producing isolate, *A. baumannii* ST730, in the Institut Pasteur database. ST730 is a single-locus variation of ST79 (CC79), responsible for the spread of *bla*_OXA-23_ in Latin America (Chagas et al. 2014, Vasconcelos et al. 2015). It is worrisome that the emergent OXA-72-producing clones share the same phylogenetic origin with the clones harbouring *bla*_OXA-23_ gene, since the latter demonstrated a remarkable capacity of dissemination and maintenance along the years in Brazilian hospitals (Pagano et al. 2015). The results presented in this study highlight the importance of monitoring the spread of successful clones associated with the dissemination of *A. baumannii* carrying *bla*OXA in Brazil by molecular epidemiology methods such as MLST.
